# Supplementary material for: Sorting at embryonic boundaries requires high heterotypic interfacial tension
Source: Nat Commun. 2017 Jul 31;8:157. doi: 10.1038/s41467-017-00146-x (PMC5537356; doi:10.1038/s41467-017-00146-x)
Supplement: Supplementary file 2 — Supplementary Software 1 [file 41467_2017_146_MOESM2_ESM.zip › PottsModel/SrcPottsModel/doc/model/HalfLine.html]

HalfLine


JavaScript is disabled on your browser.


Skip navigation links


- Overview
- Package
- Class
- Use
- Tree
- Deprecated
- Index
- Help

- Prev Class
- Next Class

- Frames
- No Frames

- All Classes

- Summary:
- Nested |
- Field |
- Constr |
- Method

- Detail:
- Field |
- Constr |
- Method


model

## Class HalfLine

- java.lang.Object
- - model.HalfLine

- All Implemented Interfaces:
  :   Vector

  ---

    

  ```
  public class HalfLine
  extends java.lang.Object
  implements Vector
  ```

- - ### Constructor Summary

    Constructors

    Constructor and Description

    `HalfLine()`

    `HalfLine(Coordinates c1,
    Coordinates c2)`

    `HalfLine(HalfLine pHalfLine)`

    `HalfLine(java.awt.Point pOrigin,
    double pAngle)`

    `HalfLine(java.awt.Point pOrigin,
    java.awt.Point pEnd)`
  - ### Method Summary

    All Methods Instance Methods Concrete Methods

    | Modifier and Type | Method and Description |
    | --- | --- |
    | `double` | `getAngle()` |
    | `java.awt.Point` | `getOrigin()` |
    | `java.awt.Point` | `getTarget()` |
    | `double` | `measureAngle(java.awt.Point p)` |
    | `void` | `pointTowards(java.awt.Point pTarget)` Length is unchanged |
    | `void` | `rotate(java.awt.Point p, float angleRatio)` Move the vector towards a point by a percentage of the angle between them. |
    | `void` | `setAngle(double pAngle)` |
    | `void` | `setOrigin(java.awt.Point pOrigin)` |
    | `java.lang.String` | `toString()` |

    - ### Methods inherited from class java.lang.Object

      `equals, getClass, hashCode, notify, notifyAll, wait, wait, wait`

- - ### Constructor Detail


    - #### HalfLine

      ```
      public HalfLine()
      ```


    - #### HalfLine

      ```
      public HalfLine(HalfLine pHalfLine)
      ```


    - #### HalfLine

      ```
      public HalfLine(java.awt.Point pOrigin,
                      double pAngle)
      ```


    - #### HalfLine

      ```
      public HalfLine(Coordinates c1,
                      Coordinates c2)
      ```


    - #### HalfLine

      ```
      public HalfLine(java.awt.Point pOrigin,
                      java.awt.Point pEnd)
      ```
  - ### Method Detail


    - #### getAngle

      ```
      public double getAngle()
      ```

      Specified by:
      :   `getAngle` in interface `Vector`


    - #### getOrigin

      ```
      public java.awt.Point getOrigin()
      ```

      Specified by:
      :   `getOrigin` in interface `Vector`


    - #### setOrigin

      ```
      public void setOrigin(java.awt.Point pOrigin)
      ```

      Specified by:
      :   `setOrigin` in interface `Vector`


    - #### setAngle

      ```
      public void setAngle(double pAngle)
      ```


    - #### pointTowards

      ```
      public void pointTowards(java.awt.Point pTarget)
      ```

      Length is unchanged

      Specified by:
      :   `pointTowards` in interface `Vector`

      Parameters:
      :   `pTarget` -


    - #### getTarget

      ```
      public java.awt.Point getTarget()
      ```

      Specified by:
      :   `getTarget` in interface `Vector`


    - #### toString

      ```
      public java.lang.String toString()
      ```

      Overrides:
      :   `toString` in class `java.lang.Object`


    - #### measureAngle

      ```
      public double measureAngle(java.awt.Point p)
      ```

      Specified by:
      :   `measureAngle` in interface `Vector`


    - #### rotate

      ```
      public void rotate(java.awt.Point p,
                         float angleRatio)
      ```

      Description copied from interface: `Vector`

      Move the vector towards a point by a percentage of the angle between them.
      For example, rotating the angle by 1 will make the vector point towards p.
      Rotating the angle by 0 will do nothing. Rotating the angle by 0.5 will make the vector point
      in the middle of the old vector and the vector pointing to p.

      Specified by:
      :   `rotate` in interface `Vector`

      Parameters:
      :   `p` - The point to move the vector towards.


Skip navigation links


- Overview
- Package
- Class
- Use
- Tree
- Deprecated
- Index
- Help

- Prev Class
- Next Class

- Frames
- No Frames

- All Classes

- Summary:
- Nested |
- Field |
- Constr |
- Method

- Detail:
- Field |
- Constr |
- Method
